# Retraction Note: Carbon nano-onion-mediated dual targeting of P-selectin and P-glycoprotein to overcome cancer drug resistance

**DOI:** 10.1038/s41467-026-74646-0

**Published:** 2026-06-24

**Authors:** Hai Wang, Yutong Liang, Yue Yin, Jie Zhang, Wen Su, Alisa M. White, Bin Jiang, Jiangsheng Xu, Yuntian Zhang, Samantha Stewart, Xiongbin Lu, Xiaoming He

**Affiliations:** 1 Fischell Department of Bioengineering, University of Maryland, College Park, MD 20742 USA; 2 CAS Key Laboratory for Biomedical Effects of Nanomaterials & Nanosafety, CAS Center for Excellence in Nanoscience, National Center for Nanoscience and Technology, 100190 Beijing, China; 3 University of Chinese Academy of Sciences, 100049 Beijing, China; 4 Department of Medical and Molecular Genetics and Melvin and Bren Simon Cancer Center, Indiana University School of Medicine, Indianapolis, IN 46202 USA; 5 Marlene and Stewart Greenebaum Comprehensive Cancer Center, University of Maryland, Baltimore, MD 21201 USA; 6 Robert E. Fischell Institute for Biomedical Devices, University of Maryland, College Park, MD 20742 USA

Retraction to: *Nature Communications* (2021) **12**:312; 10.1038/s41467-020-20588-0; article published online 12 January 2021

This paper is being retracted after a joint research misconduct investigation conducted by Indiana University, The Ohio State University, and the University of Maryland, College Park found that Figure 6b and Supplementary Fig. 24b contain irregularities. The Editor has therefore lost confidence in this paper.

Hai Wang and Xiaoming He disagree with this retraction.

Bin Jiang, Yutong Liang, Xiongbin Lu, Samantha Stewart, Wen Su, Alisa White, Jiangsheng Xu, Yue Yin, Yuntian Zhang and Jie Zhang did not respond to correspondence from the Publisher about this retraction.

